# Association between systemic immune-inflammation index and post-stroke depression: a cross-sectional study of the national health and nutrition examination survey 2005–2020

**DOI:** 10.3389/fneur.2024.1330338

**Published:** 2024-03-15

**Authors:** Mingzhu Wang, Chengchao Peng, Tingting Jiang, Qiongfang Wu, Danping Li, Min Lu

**Affiliations:** ^1^Department of Rehabilitation Medicine, Tongji Hospital, Tongji Medical College, Huazhong University of Science and Technology, Wuhan, China; ^2^Department of Critical Care Medicine, Union Hospital, Tongji Medical College, Huazhong University of Science and Technology, Wuhan, China

**Keywords:** systemic immune-inflammation index (SII), NHANSE, emotional disorders, post-stroke depression (PSD), cross-sectional study

## Abstract

**Background:**

Less research has linked the Systemic Immune Inflammatory Index (SII) with post-stroke depression (PSD). This study aims to look at any potential connections between SII and PSD.

**Methods:**

The National Health and Nutrition Examination Survey (NHANES), conducted in a population that embodied complete SII and stroke data from 2005 to 2020, was used to perform the current cross-sectional survey. A fitted smoothed curve was used to depict the nonlinear link between SII and PSD, and multiple linear regression analysis demonstrated a positive correlation between SII and PSD.

**Results:**

Multiple linear regression analysis showed that SII and PSD were markedly related [1.11(1.05, 1.17)]. Interaction tests showed that the association between SII and PSD was not statistically different between strata, and age, sex, BMI, income poverty ratio, education level, smoking status, diabetes mellitus, coronary heart disease, and heart failure did not have a significant effect on this positive association (*p* > 0.05 for interaction). In addition, a nonlinear association between SII and PSD was found using a two-stage linear regression model.

**Conclusion:**

The results of our research support the existence of a significant positive correlation between SII levels and PSD. Further prospective trials are required to comprehend SII, which is for the PSD thoroughly.

## 1 Introduction

Stroke is the second leading cause of death globally and the leading cause of disability due to its high morbidity and mortality (1, 2). It is widely known that a wide variety of functional impairments is prompted by stroke, such as cognitive impairment, motor dysfunction, and psychiatric disorders (3, 4).

Depression is the most common and severe neuropsychiatric complication after stroke (5), with a higher prevalence of post-stroke depression (PSD) of about 18–30%, and is considered an essential aspect affecting the recovery process of stroke patients (6). Patients with post-stroke depression are more prone to motor and cognitive dysfunction, thus decreasing the quality of life of patients (7) and hindering their reintegration into society and life (8, 9). Further research is still underway to explore the etiology, pathophysiologic mechanisms, risk factors, management, prevention, and treatment of depression after stroke (10, 11).

Depressive disorders are difficult to explain by a single etiology and mechanism; symptoms vary from person to person, and multiple etiologies and pathophysiologic mechanisms may be involved in different patients. Existing evidence supports that alterations in neurotransmitters, inflammatory responses, neuroendocrine activation, neuronal plasticity, and neurotrophic factors may play a role in identifying disease processes (12).

Immunity and inflammation are critical factors in stroke pathobiology, and inflammation is usually considered a tissue damage response. However, there is a growing view that inflammation affects the brain long after a stroke, especially in strokes caused by arterial occlusion or ischemic stroke (13, 14). Notably, a wealth of studies have shown that increased amounts of some markers in the blood system that respond to the level of inflammation, such as interferon-γ (IFN-γ), IL-2, IL-6, IL-8 and tumour necrosis factor (TNF-α), also play a role in the pathophysiological causes of depression (15). Therefore, several researchers have conducted experiments with animal subjects to verify whether inflammatory processes are involved in the generation of PSD disorders, and this has been supported by evidence (16–18).

In recent years, a new composite index, the Systemic Immune Inflammatory Index (SII), has been pointed out to be sensitive to the inflammatory response as a haematological marker of classical systemic inflammation. The concept of SII was first proposed by Hu et al. (19), who found that SII had a more sensitive predictive ability for hepatocellular carcinoma than one or two cell subsets. The numerator of the formula for SII is platelet count × neutrophil count, and the denominator is lymphocyte count. Inflammation is an essential component of many diseases. SII as an indicator of inflammation is a hot research topic today due to the fact that these cell counts can be obtained in the hospital with a simple lab test. Multiple investigations have additionally established the link between SII and other illnesses such as depression (20), hyperlipidemia (21), coronary heart disease (22), and stroke (23, 24).

A comprehensive review and meta-analysis showed that the SII levels of the groups with poor outcomes, such as mortality and moderate–severe stroke, were significantly greater than those of the groups with positive outcomes, such as survival and minor stroke (23). Yining Xiao and colleagues observed that an increased SII was linked to the severity of cerebral small vessel disease (CSVD) burden, suggesting that a heightened SII might contribute to cognitive impairment by exacerbating the burden of CSVD (25). Several researchers have explored the relationship between SII and mental illness, particularly depression (26, 27). And J. Wang and colleagues, at the core of their research, people with diabetes with depression gain significantly higher levels of SII than people with diabetes without depression. This finding suggests that elevated SII means that people with diabetes are at increased risk for depression. In a prospective stroke cohort study (28), elevated SII, platelet-to-lymphocyte (PLR), derived neutrophil-to-lymphocyte ratio (dNLR), and neutrophil-tolymphocyte (NLR) metrics were associated with the development of PSD and, in particular, were strongly associated with significantly elevated levels of SII on admission. Thus, SII may provide a new diagnostic strategy for early identification of post-stroke depression (29).

As we know, relatively few articles have been published to date exploring the relationship between SII and post-stroke depression. Only one prospective study supports the close correlation between them. However, the study population was relatively small, and further validation based on large samples is still needed in this direction. Therefore, to determine if the SII is related to the beginning, development, and prognosis of patients with PSD in adult US participants, we conducted a cross-sectional investigation with 33683 people. We hypothesized that a strong correlation exists between PSD and SII. SII holds promise as a predictor of involvement in the early identification of PSD, thus helping physicians to make an early diagnosis of PSD and develop rational interventions.

## 2 Methods

### 2.1 Study population

**Figure 1:**
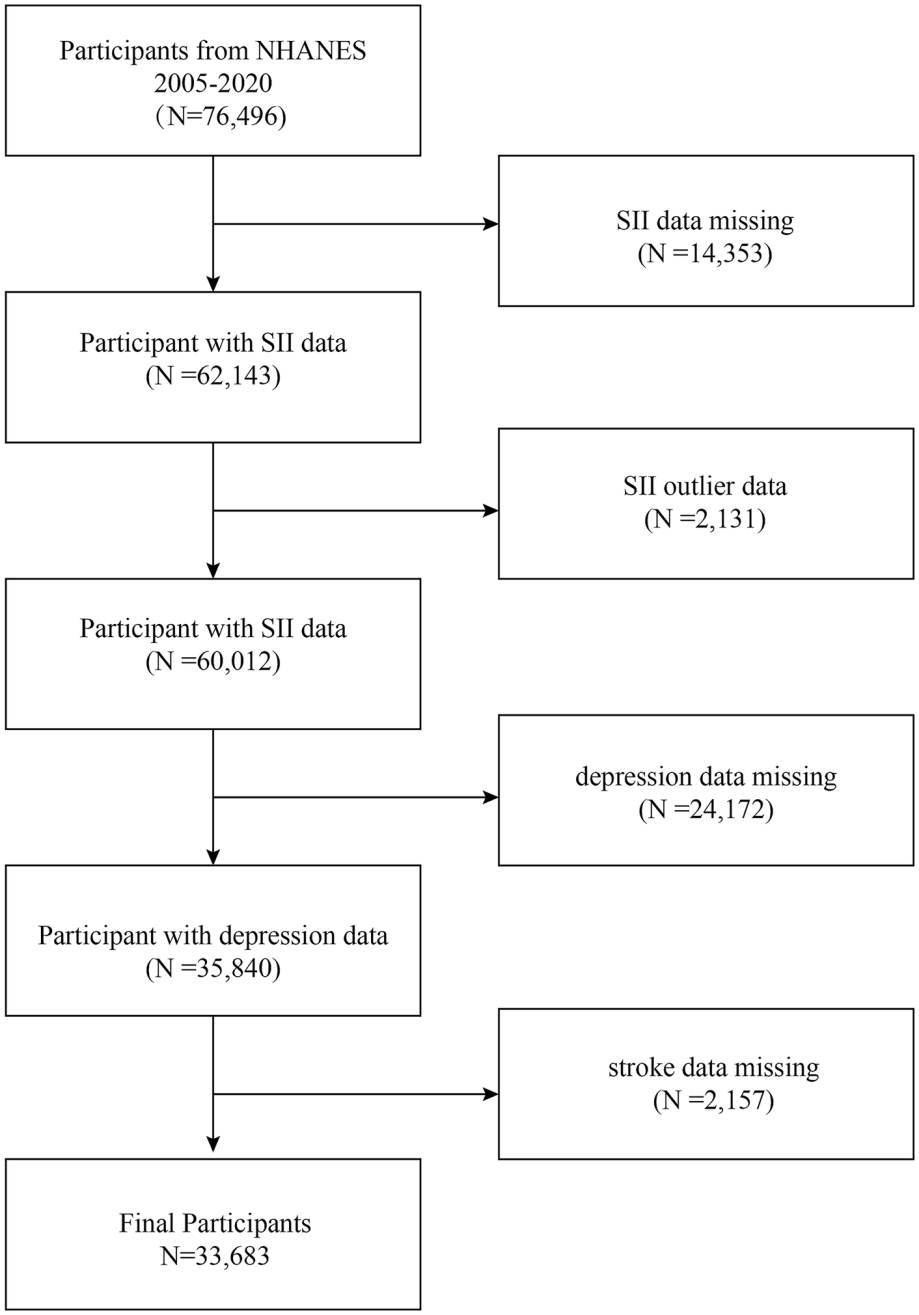
Flowchart of participant selection. NHANES, National Health and Nutrition Examination Survey.

The National Health and Nutrition Examination Survey (NHANES) was sponsored by the Centers for Disease Control and Prevention (CDC) of the United States as a population-based, cross-sectional research study to evaluate the health and nutritional status of adults and children in the country (30). It is free of charge through the Centers for Disease Control and Prevention National Center for Health Statistics (NCHS)^1^ (31). In this study, data from eight two-year cycles of NHANES (2005–2020) were combined to obtain a total of 76,496 eligible individuals. Firstly, we needed to exclude missing values for exposure factors and outcome variables; in the first step, we screened out 14353 participants with missing SII values and 2131 participants with extreme SII values and excluded them; in the second step, we removed 24172 participants with missing depression scores, and finally, 2157 participants with missing data from the stroke questionnaire were eliminated, leaving 33683 participants in this study. Figure 1 illustrates the process of sample selection.

### 2.2 Assessment of depression

With the help of the Patient Health Questionnaire (PHQ-9), a validated 9-entry patient health questionnaire, depressive symptoms were evaluated (32). It asked how often a person has experienced depressive symptoms in the past two weeks (33). Each item had four response options representing four different levels of response: “not at all,” “a few days,” “half a day,” and “almost every day.” These four responses were scored on a scale of 0 to 3 on a low to high scale, and the final tally of scores for the nine questions ranged from 0 to 27 combined. In this study, the validity and sensitivity of the PHQ-9 was 88%, with scores greater than or equal to 10 indicating a tendency toward depression (34).

### 2.3 SII and covariates

The exposure variable in our study was SII. The NHANES data website provided some laboratory data on the values that should be used in the calculation of SII, which were obtained using the laboratory method of complete blood count, which used an automated haematology analyzer to assess the counts of lymphocytes, neutrophils, and platelets in the peripheral blood and is expressed as ×10^3^ cells/μL (19, 35–37). SII was calculated by a simple multiply and divide operation, with the numerator of the formula being the platelet count × neutrophil count and the denominator being the lymphocyte count (38). Several covariates were included in the final analyses including age, sex (male or female), ethnicity (non-Hispanic white, black, Mexican American, other Hispanic, or other ethnicities), educational attainment (less than high school, high school or more than high school), income-to-poverty ratio (0 to 1.5, 1.5 to 3.5, >3.5), body mass index (<18.5, 18.5 to <25, 25 to <30, or ≥ 30 kg/m^2^) (20), current smoking (yes or no), alcohol use (yes or no), diabetes mellitus (yes or no), hypertension (yes or no), coronary heart disease (yes or no), congestive heart failure (yes or no), and low-density lipoprotein (LDL). LDL (continuous), HDL (continuous), total cholesterol (continuous), triglycerides (continuous), and uric acid (continuous) (21, 23, 29). These covariates might be confounders associated with SII and poststroke depression.

### 2.4 Statistical analysis

Statistical analyses for this study were performed using R Studio (version 4.2.2) and EmpowerStats (version 2.0). Means are expressed as standard deviation (SD) for continuous variables and as proportions for categorical variables; SII was categorized into quartiles according to their values, arranged from low to high as Q1, Q2, Q3, and Q4, representing different intervals, respectively. When categorized by SII quartiles (continuous variables), differences between subjects were analyzed using weighted t-tests or chi-square tests. We assessed the odds ratios (ORs) and 95% confidence intervals (CIs) for the association of SII with poststroke depression using multivariate logistic regression models. In the multivariate logistic regression analysis model, we constructed three models; model 1 was unadjusted for covariates, and model 2 was adjusted for age, sex, and race. Model 3 adjusted for age, sex, race, income-to-poverty ratio, education level, body mass index, coronary heart disease, congestive heart failure, and diabetes. We were adjusting variables performed simultaneous smoothed curve fitting. It was considered statistically significant when *p* < 0.05. A weighting strategy was used to reduce the high volatility of the data set.

## 3 Results

### 3.1 Baseline characteristics of participants

For this study, a total of 33,683 eligible individuals with a mean age of (49.82 ± 17.74) years were enlisted, of which 50.98%, or nearly 2% more females than males, were female. The majority of participants were non-Hispanics, who made up the majority of the participant population. A mean SII concentration of 493.55 was measured. Additionally, 242 patients (0.72%) reported post-stroke depression.

Table 1 presents the quartiles of SII as a columnar stratification variable, while this table also includes all clinical characteristics associated with the subjects. Differences between SII quartiles were statistically significant (*p* < 0.05) for income poverty ratios, BMI, alcohol consumption, smoking, diabetes, coronary heart disease, and heart failure. Compared to those representing the lowest quartile of SII levels (Q1), those in the highest quartile (Q4) had income poverty ratios distributed mainly in the range of 1.5–3.5; more people in this study’s population responded to the survey on smoking status that they were still smoking now; and more people had diabetes, coronary heart disease, heart failure, and post-stroke depression. In contrast to higher levels of BMI, PHQ-9 scores, total cholesterol, LDL cholesterol, triglycerides, LSM, and SII, the levels of direct HDL cholesterol and uric acid were lower.

**Table 1:** Weighted characteristics of the study population based on SII quartiles.

		SII quartiles			*p*-value
	Q1	Q2	Q3	Q4	
	*N* = 8420	*N* = 8421	*N* = 8421	*N* = 8421	
Age,(years)	49.66 ± 17.74	49.98 ± 17.81	49.75 ± 17.72	49.90 ± 17.67	0.663
Gender (%)					0.146
Male	4121 (48.94%)	4057 (48.18%)	4207 (49.96%)	4128 (49.02%)	
Female	4299 (51.06%)	4364 (51.82%)	4214 (50.04%)	4293 (50.98%)	
Race (%)					0.771
Mexican American	1278 (15.18%)	1296 (15.39%)	1292 (15.34%)	1270 (15.08%)	
Other Hispanic	831 (9.87%)	805 (9.56%)	818 (9.71%)	835 (9.92%)	
Non-Hispanic White	3603 (42.79%)	3612 (42.89%)	3591 (42.64%)	3605 (42.81%)	
Non-Hispanic Black	1772 (21.05%)	1784 (21.19%)	1853 (22.00%)	1842 (21.87%)	
Other race	936 (11.12%)	924 (10.97%)	867 (10.30%)	869 (10.32%)	
Education (%)					0.187
Less than high school	2044 (24.30%)	1977 (23.49%)	1898 (22.55%)	1995 (23.71%)	
High school	1903 (22.62%)	1970 (23.41%)	1995 (23.70%)	1925 (22.88%)	
More than high school	4465 (53.08%)	4469 (53.10%)	4524 (53.75%)	4494 (53.41%)	
Income to poverty ratio (%)					<0.001
0–1.5	3002 (35.65%)	2823 (33.52%)	2587 (30.72%)	2567 (30.48%)	
1.5–3.5	3124 (37.10%)	3203 (38.04%)	2587 (30.72%)	3380 (40.14%)	
>3.5	2294 (27.24%)	2395 (28.44%)	2514 (29.85%)	2474 (29.38%)	
BMI (%)					<0.001
0–25	2533 (30.08%)	2388 (28.36%)	2201 (26.14%)	2149 (25.52%)	
25–30	2999 (35.62%)	2930 (34.79%)	2844 (33.77%)	2759 (32.76%)	
>30	2888 (34.30%)	3103 (36.85%)	3376 (40.09%)	3513 (41.72%)	
Smoking status (%)					<0.001
Smoking now	1239 (34.58%)	1297 (35.76%)	1392 (36.71%)	1556 (39.13%)	
Smoking former	360 (10.05%)	312 (8.60%)	327 (8.62%)	306 (7.70%)	
Never	1984 (55.37%)	2018 (55.64%)	2073 (54.67%)	2114 (53.17%)	
Diabetes (%)					0.001
Yes	1275 (15.15%)	1257 (14.93%)	1258 (14.95%)	1415 (16.82%)	
No	7141 (84.85%)	7162 (85.07%)	7156 (85.05%)	6997 (83.18%)	
Coronary heart disease (%)					0.047
Yes	330 (3.93%)	298 (3.55%)	357 (4.26%)	361 (4.30%)	
No	8070 (96.07%)	8094 (96.45%)	8031 (95.74%)	8026 (95.70%)	
Congestive heart failure (%)					<0.001
Yes	257 (3.06%)	210 (2.50%)	253 (3.01%)	333 (3.96%)	
No	8150 (96.94%)	8194 (97.50%)	8140 (96.99%)	8071 (96.04%)	
Post stroke depression (%)					
Yes	45 (0.53%)	51 (0.61%)	54 (0.64%)	92 (1.09%)	<0.001
No	8375 (99.47%)	8370 (99.39%)	8367 (99.36%)	8329 (98.91%)	
PHQ-9 scores	3.10 ± 4.38	3.12 ± 4.58	3.19 ± 4.37	3.65 ± 4.89	<0.001
Alcohol (days)	2.49 ± 2.12	2.54 ± 2.19	2.54 ± 2.33	2.52 ± 2.27	0.855
LDL (mg/dl)	112.65 ± 24.75	113.56 ± 25.09	113.36 ± 23.69	112.97 ± 23.88	0.020
HDL (mg/dl)	53.78 ± 16.35	52.76 ± 15.81	52.64 ± 16.07	52.95 ± 15.76	<0.001
Total cholesterol (mg/dl)	190.67 ± 41.19	192.78 ± 41.33	193.55 ± 41.29	192.72 ± 41.46	<0.001
Triglyceride (mg/dl)	111.21 ± 80.27	113.62 ± 69.77	113.81 ± 66.79	113.27 ± 60.95	<0.001
Uric acid (mg/dl)	5.46 ± 1.40	5.45 ± 1.41	5.44 ± 1.41	5.42 ± 1.48	0.013

Mean ± SD for continuous variables: the *p*-value was calculated by a weighted linear regression model. % for categorical variables: the *p*-value was calculated by a weighted chi-square test. Q, quartile; BMI, body mass index.

### 3.2 Association between SII and post-stroke depression

The issue of negligible effect values was resolved by implementing SII/100 to multiply the effect values by 100. The outcomes of the multivariate regression analysis between SII/100 and depressive disorders following a stroke are shown in Table 2. Model 1 (1.11 (1.05, 1.17)), Model 2 (1.11 (1.05, 1.17)), and Model 3 (1.09 (1.03, 1.16)) all exhibited significant relationships between the two. In model 2, sensitivity analyses were performed on SII quartiles, with or of 1.0, 1.13 (0.76, 1.69), 1.20 (0.81, 1.79), 2.06 (1.44, 2.95) for Q1, Q2, Q3 and Q4, respectively. Participants in quartile 4 had an increased risk of post-stroke depression by 106% (*p* < 0.0001) compared with quartile 1.

**Table 2:** The association between SII and post-stroke depression.

	Crude model (Model 1)	Partially adjusted model (Model 2)	Fully adjusted model (Model 3)
	OR (95% CI) *p*-value	OR (95% CI) *p*-value	OR (95% CI) *p*-value
SII/100	1.11 (1.05, 1.17) ***	1.11 (1.05, 1.17) ***	1.09 (1.03, 1.16) **
SII quartiles			
Quartile 1	1.0	1.0	1.0
Quartile 2	1.13 (0.76, 1.70)	1.13 (0.76, 1.69)	1.19 (0.78, 1.80)
Quartile 3	1.20 (0.81, 1.79)	1.20 (0.81, 1.79)	1.28 (0.85, 1.92)
Quartile 4	2.06 (1.44, 2.94) ***	2.06 (1.44, 2.95) ***	2.02 (1.39, 2.93) ***
*p* for trend	<0.0001	<0.0001	<0.0001

Model 1, no covariates were adjusted. Model 2, age, gender, and race were adjusted. Model 3, age, gender, race, income to poverty ratio, education level, BMI, Coronary heart disease, Congestive heart failure and diabetes were adjusted. 95% CI, 95% confidence interval; OR, odds ratio; SII, systemic immunity-inflammation index. * *p* < 0.05, ** *p* < 0.01, *** *p* < 0.001; a *p* < 0.05 was considered statistically significant.

**Figure 2:**
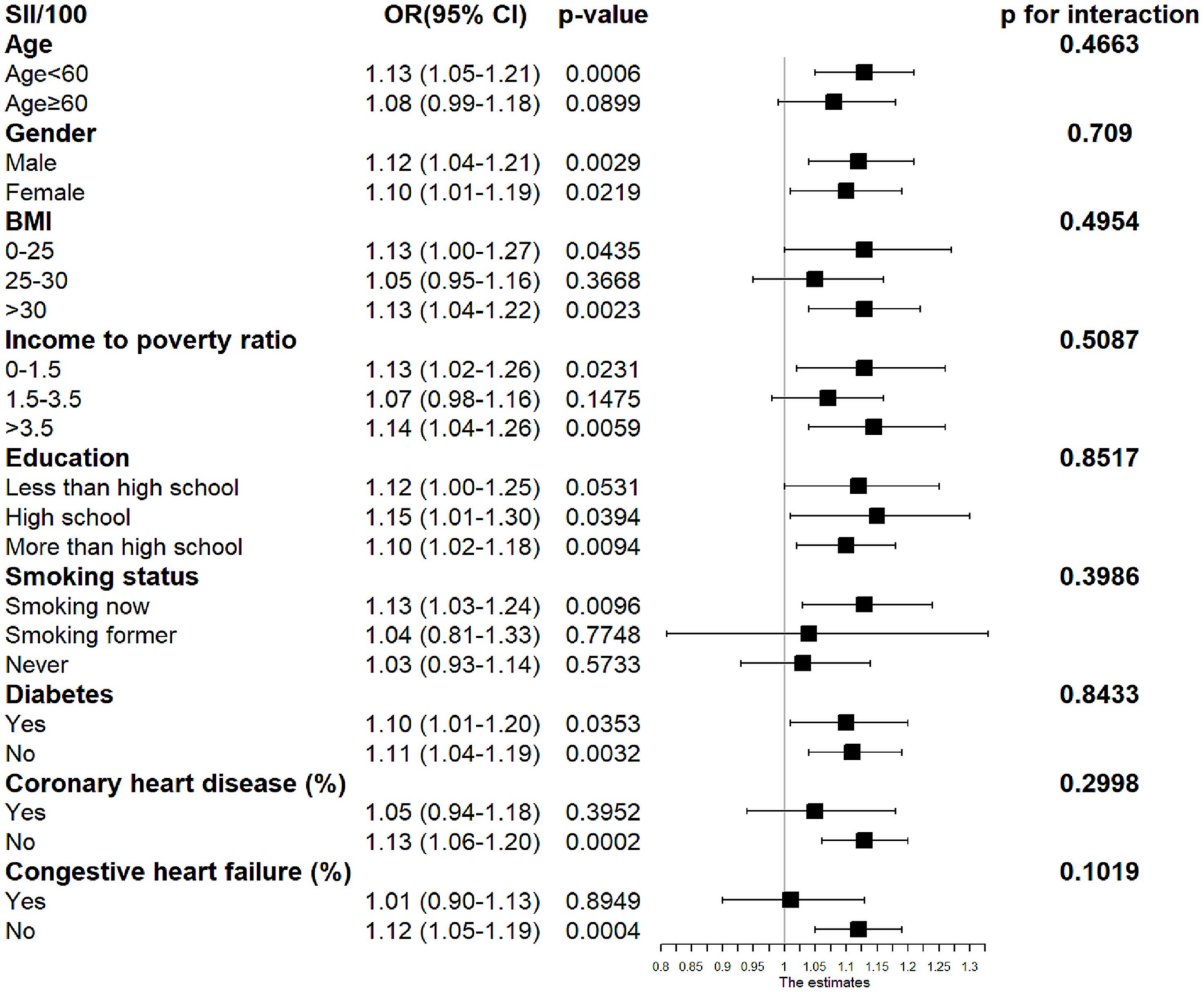
Subgroup analysis for the association between SII and post-stroke depression.

Further subgroup analyses showed that the relationship between SII and PSD was inconsistent, as Figure 2 shows. The results showed that SII was strongly linked with post-stroke depression (*p* < 0.05), with gender, education level, and diabetes mellitus as stratification factors for subgroup analysis. Age, gender, income poverty, education level, body mass index, smoking status, diabetes mellitus, coronary artery disease, and heart failure did not significantly affect this positive correlation, according to interaction tests, which revealed that the relationship between SII and post-stroke depression did not statistically differ between strata (*p* > 0.05 for interaction tests).

**Figure 3:**
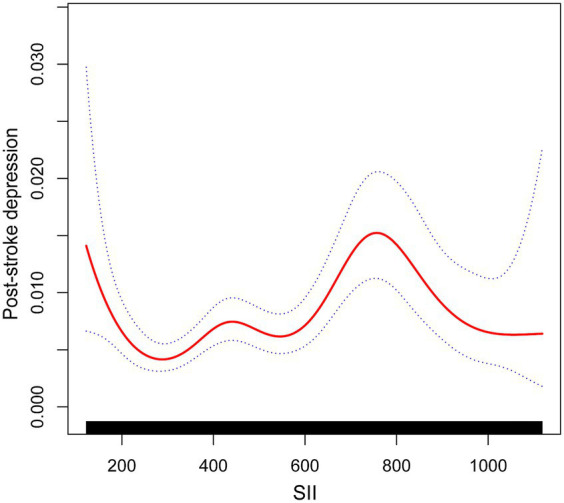
The association between SII and post-stroke depression. The solid red line represents the smooth curve fit between variables. Blue bands represent the 95% confidence interval from the fit.

Following that, smoothed curve fitting was used to depict the nonlinear association between SII and PSD (Figure 3). Gender, race, age, income-poverty ratios, education level, diabetes, BMI, coronary artery disease, and heart failure were among the adjusted variables. The nonlinear relationship between SII and post-stroke depression was described by smooth curve fitting.

## 4 Discussion

In a representative study sample of U.S. adults, after cross-sectional analysis, we found that SII levels were positively associated with the development of post-stroke depression. In addition, the highest quartile of SII was associated with an increased incidence of PSD compared with the lowest quartile of SII. The results of subgroup analyses suggest that this association is similar across populations. The study also found a nonlinear relationship between SII and PSD, suggesting that high SII levels may be an independent risk factor for PSD.

As far as we know, this was the first time researchers have conducted an analysis of the connection between SII and post-stroke depression using the NHANES database. Prior to this, according to our findings, some studies have reported an association between SII and stroke prognosis, mood disorders such as depression, and poststroke cognitive impairment (29, 39, 40). A meta-analysis of the association between SII and clinical outcomes in stroke patients noted that in 19 retrospective studies that met the inclusion criteria, the levels of SII were much higher in the poor regression, death, and moderate-to-severe stroke groups than in the excellent regression, survival, and mild stroke groups, respectively (23). SII can be used to predict poor outcomes and high mortality after stroke. Similarly, a prospective cohort study showed that SII was strongly associated with poststroke severity, whereas platelet-albumin-bilirubin was not associated with stroke severity (41).

Many epidemiologic studies have demonstrated the involvement of inflammation in the development of depression. In a multicenter cross-sectional study involving 338 hospitalized TB patients, approximately half of the patients had anxiety and/or depression symptoms. Patients with these symptoms demonstrated a more robust inflammatory response and a worse cellular immune status than those without them (26). Similarly, in a study that included 2566 diabetic patients, 13.3% were diagnosed with depression, and high SII was recognized as an independent risk factor for depression in diabetes. This confirms our finding that high SII levels are associated with an increased risk of depression (28).

Previous research indicated that responses to inflammation in many central and peripheral systems may be biological factors contributing to PSD (42, 43). During the acute phase of ischemic stroke (minutes to hours), infiltration of inflammatory cells, including granulocytes (neutrophils), T cells, monocytes/macrophages, and other cells in the peripheral circulation, infiltrate the ischemic brain areas (44–46). Subsequently, damaged tissues rapidly release oxygen free radicals (ROS) and inflammatory mediators (including cytokines and chemokines). These substances induce the expression of adhesion molecules by endothelial cells and leukocytes in the brain, which prompts peripheral leukocytes to adhere to and traverse the endothelium, directly triggering necrosis and apoptosis in the ischemic region (47, 48). Infiltrating neutrophils release free radicals, cytokines (like IL-6), chemokines (like MCP-1), protein hydrolases, and immunoglobulins during the subacute phase (which lasts for hours to days). They also stimulate/activate matrix metalloproteinase 9 (mostly MMP-9) and protein hydrolases, which worsen the inflammatory response in the brain by causing more broad cellular activation and leukocyte infiltration. In addition, platelets also play an essential role in the inflammatory response after stroke, where platelets aggregate at the site of the lesion. Activated platelets tend to roll and adhere to endothelial cells in the intima of blood vessels from the flowing bloodstream, altering their functional properties and leading to increased neutrophil chemotaxis (49).

These markers of the inflammatory response affect neurotransmission, leading to a legacy of severe dysfunction in the brain, such as mood disorders (depression), cognitive impairment, etc. (50, 51). An out-of-control inflammatory response following stroke can produce damage to the blood–brain barrier, affecting its permeability and making it easy for leukocytes to penetrate it, which has been associated with causing a variety of adverse stroke prognoses in the brain, including pathologic cerebral oedema, hemorrhagic transformation, and higher mortality. A prospective stroke cohort study found that changes in cell counts in the circulating blood of patients with acute ischemic stroke could be represented by composite indices of the inflammatory response, such as SII, dNLR, PLR, and NLR, and found that the development of PSD was inextricably linked to elevated levels of these inflammatory composite indices, exceptionally high SII. Our study observed similar results, suggesting that high SII was positively associated with poststroke depression risk was positively associated (29).

Previous studies have confirmed that early elevation of inflammatory factors such as NLR and PLR measured in peripheral blood on admission to the hospital may provide physicians with some information to facilitate early diagnosis of PSD (52–54). Our study aligns with this perspective, and SII adds a platelet count to the NLR formula, which is more generalized than NLR and PLR.

Currently, clinical treatment of post-stroke depression (PSD) suggests a combination of psychotherapy, medication, and rehabilitation training to achieve optimal results. Although there are no precise research results to support the application of SII in PSD treatment, the correlation between the two suggests that detecting SII levels may guide immunotherapy, anti-inflammatory therapy, and antidepressant therapy for PSD and improve the clinical efficacy of PSD patients. Inflammation may be an essential disease-modifying factor contributing to susceptibility to depression, and controlling inflammation may be beneficial to treatment (55). Some researchers have suggested that long-term use of antidepressant medications may have a therapeutic effect by reducing the appearance of LPS-induced pathological behaviors that resemble depressive symptoms (manifested as reduced activity, such as reduced eating) (56). A meta-analysis of 36 randomized controlled trials (10,000 patients) (57) found antidepressant effects with monotherapy or in combination with anti-inflammatory drugs. Anti-inflammatory drugs such as cytokine inhibitors (58), statins (59), glucocorticoids (60), and nonsteroidal anti-inflammatory drugs (61) have been successfully studied with antidepressants (62). Therefore, antidepressants may also be beneficial in combination with anti-inflammatory medications.

In terms of clinical management of depressed patients after stroke, we can provide early detection and risk stratification of stroke patients based on SII levels and individualized management and treatment based on the degree of inflammatory response. SII levels are also monitored to assess the effectiveness of interventions. Regular monitoring of SII levels during long-term follow-up may also be helpful in early detection of potential recurrence or the emergence of new depressive symptoms, which can lead to timely adjustment of the treatment program. Discovering the correlation between SII and PSD is an essential clinical guide for screening, etiologic research, treatment, and prognostic assessment of PSD. And it provides a more refined and targeted approach to clinical management. Incorporating SII into the evaluation and treatment plan is expected to improve the clinical outcomes of patients. This is a direction well worth in-depth study.

Our study has several limitations; first, it was difficult for us to obtain a clear causal relationship between the exposure factors and the outcome variables due to the cross-sectional study design. Future studies still need more prospective studies to elucidate the causal relationship between SII and PSD. Second, there was no way to include all covariates for analysis because the amount of missing individual covariates was too large. Third, due to the limitations of the NHANES database, we were not able to have self-rating instruments and rater-administered tools such as the Beck Depression Inventory, the Self-Depression Scale (SDS), and the Hamilton Rating Scale for Depression (HRSD), which are more sophisticated depression assessment tools. Using only the PHQ-9 to assess the presence and severity of depression is somewhat different from the assessment of clinical depression. Forth, we should have included the population’s anti-inflammatory drug use history and need to know the use of antiplatelet, which may have affected our results. Despite these limitations, our survey has several strengths. First, the NHANES data, a national population-based sample obtained using standardized protocols. Second, the reliability and representativeness of our study are enhanced by the large sample size and appropriate correction for covariates, as well as subgroup analyses. Third, the potential value of measuring systemic inflammatory biomarkers in identifying individuals at risk for depression in the stroke population is emphasized and has the potential to provide new diagnostic and treatment options for depression.

## 5 Conclusion

This cross-sectional study based on the NHANES database demonstrated a significant positive association between SII levels and post-stroke depression. However, the findings do not yet conclusively indicate a causal association between SII and PSD, which should be confirmed in further prospective studies.

## Data availability statement

The original contributions presented in the study are included in the article/Supplementary material, further inquiries can be directed to the corresponding author.

## Ethics statement

The studies involving human participants were reviewed and approved by National Center for Health Statistics Ethics Review Board. The patients/participants provided their written informed consent to participate in this study. The studies were conducted in accordance with the local legislation and institutional requirements. The participants provided their written informed consent to participate in this study.

## Author contributions

MW: Conceptualization, Data curation, Formal analysis, Methodology, Writing – original draft, Software. CP: Conceptualization, Methodology, Writing – original draft, Data curation, Formal analysis. TJ: Formal analysis, Writing – review & editing, Investigation, Methodology. QW: Supervision, Visualization, Writing – review & editing, Methodology, Software. DL: Supervision, Visualization, Writing – review & editing. ML: Conceptualization, Supervision, Writing – review & editing.

## Footnotes

^1^https://wwwn.cdc.gov/nchs/
